# Genome-Wide Identification and Expression Analysis of Cysteine-Rich Polycomb-like Protein (CPP) Gene Family in Tomato

**DOI:** 10.3390/ijms24065762

**Published:** 2023-03-17

**Authors:** Yaoguang Sun, Xinyi Jia, Dexia Chen, Qingjun Fu, Jinxiu Chen, Wenhui Yang, Huanhuan Yang, Xiangyang Xu

**Affiliations:** Key Laboratory of Biology and Genetic Improvement of Horticultural Crops (Northeast Region), Ministry of Agriculture and Rural Affairs, College of Horticulture and Landscape Architecture, Northeast Agricultural University, Harbin 150030, China

**Keywords:** tomato, CPP gene family, abiotic and biotic stress, gene expression

## Abstract

The cysteine-rich polycomb-like protein (CPP) gene family is a class of transcription factors containing conserved cysteine-rich CRC structural domains that is involved in the regulation of plant growth and stress tolerance to adversity. Relative to other gene families, the CPP gene family has not received sufficient attention. In this study, six *SlCPPs* were identified for the first time using the most recent genome-wide identification data of tomato. Subsequently, a phylogenetic analysis classified *SlCPPs* into four subfamilies. The analysis of *cis*-acting elements in the promoter indicates that *SlCPPs* are involved in plant growth and development and also stress response. We present for the first time the prediction of the tertiary structure of these *SlCPPs* proteins using the AlphaFold2 artificial intelligence system developed by the DeepMind team. Transcriptome data analysis showed that *SlCPPs* were differentially expressed in different tissues. Gene expression profiling showed that all *SlCPPs* except *SlCPP5* were up-regulated under drought stress; *SlCPP2*, *SlCPP3* and *SlCPP4* were up-regulated under cold stress; *SlCPP2* and *SlCPP5* were up-regulated under salt stress; all *SlCPPs* were up-regulated under inoculation with *Cladosporium fulvum*; and *SlCPP1*, *SlCPP3*, and *SlCPP4* were up-regulated under inoculation with *Stemphylium lycopersici*. We performed a virus-induced gene silencing experiment on *SlCPP3*, and the results indicated that *SlCPP3* was involved in the response to drought stress. Finally, we predicted the interaction network of the key gene *SlCPP3*, and there was an interaction relationship between *SlCPP3* and 10 genes, such as *RBR1* and *MSI1*. The positive outcome showed that *SlCPPs* responded to environmental stress. This study provides a theoretical and empirical basis for the response mechanisms of tomato in abiotic stresses.

## 1. Introduction

Tomato (*Solanum lycopersicum*), which is a representative crop species of *Solanaceae*, is a bulk horticultural crop with high yields and efficiency, and it is the most widely cultivated fruit and vegetable crop in the world [1,2]. However, various stresses encountered in nature, such as drought, cold, salt, diseases and pests, are constantly threatening the normal growth and reproduction of plants, potentially leading to large decreases in yield and quality [3,4,5]. These unfavorable factors serve as the weathervane that determines the evolutionary direction in the long-term development of plants [6]. The adaptation of plants to the drought environment is ultimately achieved by controlling the expression of relevant genes [7]. A large number of transcription factors related to drought stress regulation have been cloned, such as *DERB*/*CBF*, *ABF*, *ABI3*/*4*/*5*, *MYB*, *NAC*, etc. [8]. Under drought conditions, plants can rapidly produce the adversity hormone abscisic acid (ABA), which in turn regulates stomatal movement [9]. The regulatory mechanisms of plants to saline stress are mainly divided into: (1) signal transduction, (2) ion homeostasis, (3) changes in the content of various hormones and the response of hormone signals in plants, and (4) the regulation of gene expression [10]. Compared with other abiotic stresses, the response of plants to low temperature is very complex, and studies have shown that *H2A.Z* histone plays an important role in plant perception of external temperature changes [11]. For the genetic evolution of plant populations, plants gradually evolve over a long period of time to adapt to their environment, where the evolution of the genome is particularly important [12]. Various gene families can regulate plant perception of the environment in the genome, and they are interspersed to form a complex network system [13]. The identification and further elucidation of the effects of regulatory genes are indispensable in on-going research.

The cysteine-rich polycomb-like protein (CPP) transcription factor gene family is widespread in plants and belongs to a small group including other gene families [14]. CPP proteins usually contain CXC domains, with a conserved of CXCX4CX3YCXCX6CX3CXCX2C [15]. A variable-length linking sequence is usually found between two CXC motifs, which generally contains a conserved R sequence (RNPXAFXPK) [16]. It is customary to refer to the region consisting of these three conserved sequences as the CRC domain, which is an important marker of the CPP transcription factor gene family [17]. The CPP gene family members have been identified and analyzed in a large number of plants [18,19,20]. These CPP transcription factors are involved in processes such as the regulation of specific plant growth and development, induction of multiple hormones, and stress responses.

The CXC domain of CPP transcription factors can regulate target gene expression by binding to specific DNA sequences. *TSO1* (*AtCPP5*) is an essential gene for floral organ formation in *A. thaliana* [21]. The *TSO1* protein is an essential component of the flower-specific cellular mitosis machinery, but it does not play this specific role in other tissues [22]. Previous studies have shown that *tso1* mutants present disordered mitosis in floral organ cells, failure to form complete cell walls at the end of mitosis, a disordered phloem structure, and a rapid increase in DNA content in the nucleus, leading to morphological abnormalities in petals, stamens and carpels [23,24]. Recent studies have shown that *TSO1* and *MYB3R1* can form a conserved cell cycle regulatory module that coordinates cell proliferation and shoot- and root-specific differentiation [25]. In maize, the 13 identified *ZmCPP* genes present different expression patterns in response to abiotic stresses [19]. In studies of soybean root systems during the response to abiotic stresses, most *GmCPP* genes [18] were found to be significantly upregulated in response to high-temperature induction under drought stress conditions, suggesting that these genes are involved in the regulation of the soybean root system in response to high-temperature stress [20]. *GmCPP1* can interact with the promoter of the soybean hemoglobin gene *Gmlbc3* and is involved in the regulation of leg hemoglobin genes in symbiotic root nodules [26].

In this study, based on the most recent tomato genomic data (ITAG 4.0), we identified six *SlCPPs* for the first time. These *SlCPP* genes were systematically characterized. Based on bioinformatics analysis, we performed a comprehensive characterization of these *SlCPPs*, including (physicochemical properties, gene localization, interspecific homology, etc.). Subsequently, the protein structure of the *SlCPP* genes was predicted for the first time by using AlphaFold2 software developed by DeepMind (https://www.deepmind.com/open-source/alphafold-protein-structure-database, accessed 22 March 2022). The tissue specificity of *SlCPPs* was characterized. We also explored the expression pattern of *SlCPPs* under both abiotic (drought, cold and salt) and biotic stress (inoculation with *Cladosporium fulvum* and *Stemphylium lycopersici*) treatments, and the interaction between *SlCPP3* and other genes. Our findings provide a solid foundation for the further exploration of tomato CPP gene family functions.

## 2. Results

### 2.1. Identification and Characterization of CPP Genes in Tomato

**Figure 1:**
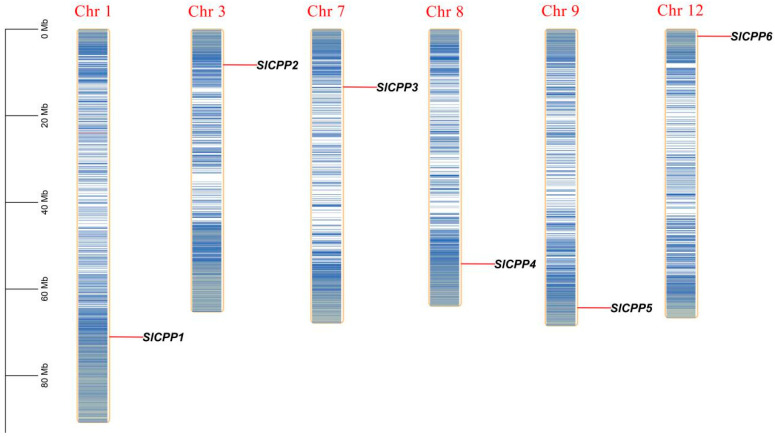
Distribution of *SlCPP* genes on tomato chromosomes. The red line indicates the gene position, and the blue line indicates the gene density.

After screening by HMMER 3.0 and validation with the SMART and CDD online tools, six *SlCPP* genes were identified, which were evenly distributed on six chromosomes in tomato (chromosomes 1, 3, 7, 8, 9 and 12) and were numbered *SlCPP1*-*6* according to their chromosomal locations. The positions of the *SlCPPs* on the chromosomes are shown in Figure 1.

The coding sequence (CDS) lengths of these *SlCPPs* ranged from 915 (*SlCPP5*) to 2922 bp (*SlCPP4*). The *SlCPPs* proteins have amino acid (aa) numbers in the range of 304 (*SlCPP5*)-973 (*SlCPP4*) aa, molecular weights in the range of 33.02 (*SlCPP5*)-104.73 (*SlCPP4*) kDa, instability indices in the range of 42.54 (*SlCPP1*)-66.02 (*SlCPP5*), and an aliphatic index ranging between 59.08 (*SlCPP5*) and 69.11 (*SlCPP4*). The results of a hydrophilicity analysis showed that all six *SlCPP* genes encoded hydrophilic proteins (negative Grand average of hydropathicity (GRAVY) values for hydrophilic groups and positive values for hydrophobic groups). The results of subcellular localization prediction indicated that all *SlCPP* genes were localized in the nucleus. Supplementary Table S1 provides more detailed information.

### 2.2. Phylogenetic Relationships and Gene Structure Analysis of SlCPPs

**Figure 2:**
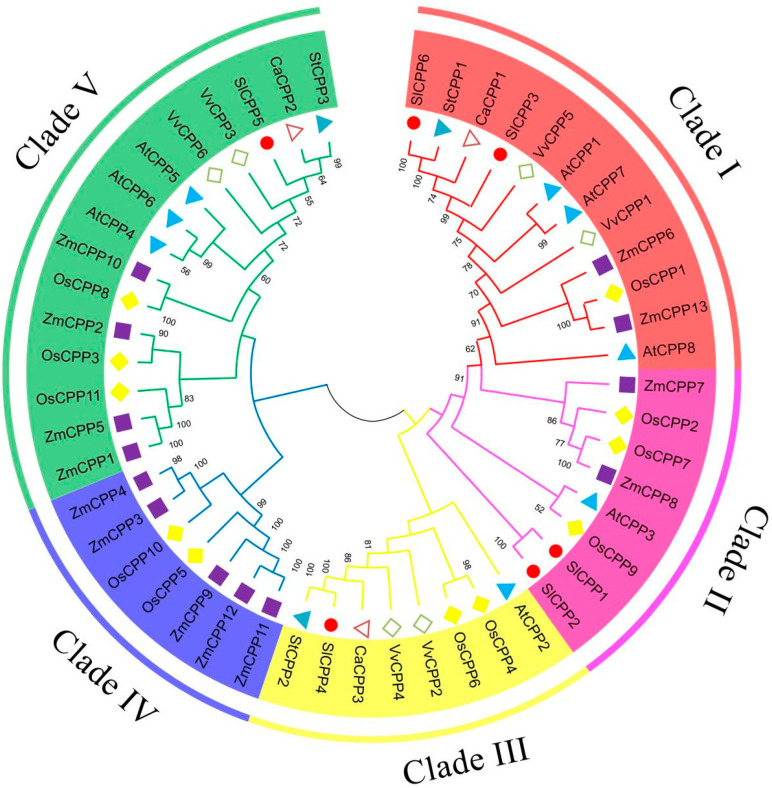
Phylogenetic tree of CPP family members from *Solanum lycopersicum* (Sl), *A. thaliana* (At), *Capsicum annuum* (Ca), *Solanum tuberosum* (St), *Zea mays* (Zm), *Vitis vinifera* (Vv) and *Oryza sativa* (Os). Different squares represent different plants, and different clades are indicated by different colors. Nodes with bootloader support values of less than 50 are not shown.

To elucidate the phylogenetic relationships of *SlCPPs* in tomato, a phylogenetic tree of the full-length protein sequences of the tomato *SlCPP* family along with the CPP gene family members of six other species was constructed and analyzed. The CPP family was classified into five clades (Clades I-V). The tomato *SlCPP* family members were distributed in four clades, among which *SlCPP6* and *SlCPP3* were distributed in Clade I, *SlCPP1* and *SlCPP2* in Clade II, and *SlCPP4* and *SlCPP5* in Clades III and V. More details are shown in Figure 2. The tomato *SlCPP* genes are more closely evolutionarily related to the members of the CPP gene families of potato and pepper, which are also members of the *Solanaceae* family.

**Figure 3:**
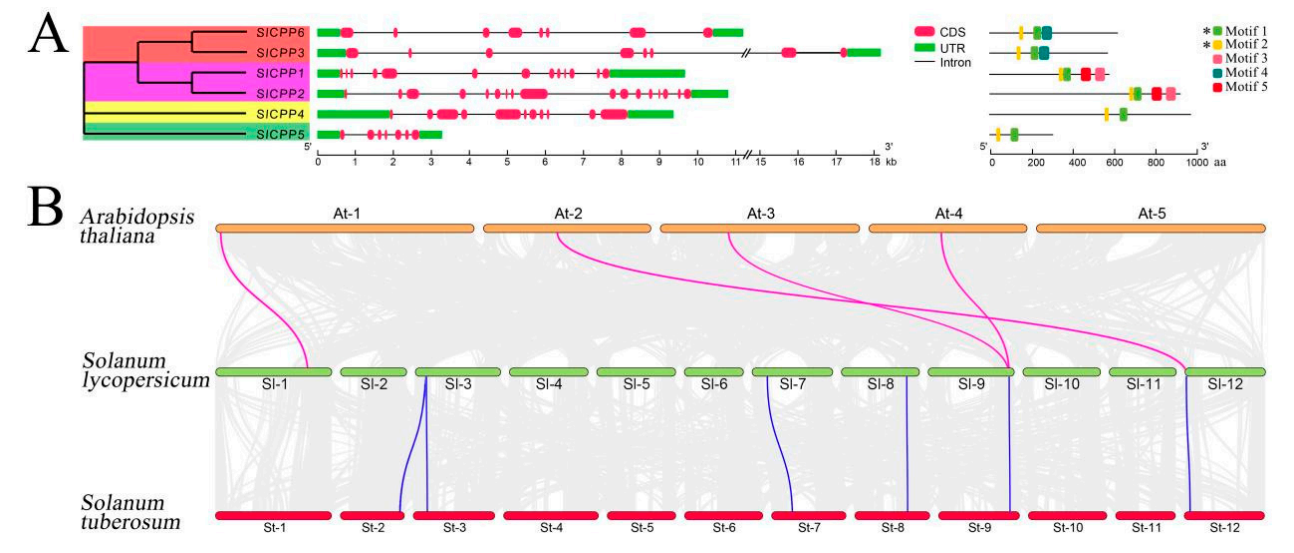
Gene structure and syntenic relationships of the CPP gene family. (**A**) Gene structure of the CPP gene family in tomato. The asterisks indicate Motifs shared by all *SlCPPs*. (**B**) Syntenic relationships between homologous *SlCPPs* of tomato and other species.

The phylogenetic tree of the six *SlCPP* genes was divided into four branches, as shown in Figure 3A. Genetic structure analysis showed that the number of exons in the *SlCPP* genes ranged from 7 (*SlCPP5*) to 17 (*SlCPP2*), with *SlCPP6* and *SlCPP3* of clade I containing eight exons. The results of conserved motifs analysis of *SlCPPs* proteins showed that all members have two CXC structural domains (Motif 1 and Motif 2, or C1 and C2) containing Cys-rich domain sequences. More intriguingly, three specific conserved motifs (motifs 3–5) are harbored in the *SlCPPs*, as shown in Supplementary Figure S1. The number, type and arrangement of the *SlCPP* gene motifs located on the same branch are similar, and the functional differences in tomato *SlCPP* genes may be due to the differences in the distribution of conserved motifs.

### 2.3. Synteny Analysis of SlCPP Genes

Synteny analysis is a critical analytical strategy in comparative genomics that plays an essential role in assessing the molecular evolutionary relationships between species [27]. Homology analysis of the *SlCPPs* between tomato and other species suggested that the CPP gene was more homologous on tomato and potato, probably because of their close kinship, as shown in Figure 3B and Supplementary Table S2. Notably, *SlCPP2* and *SlCPP5* correspond to two gene pairs present in potato and *A. thaliana*, respectively. Synteny analysis of *SlCPP* genes showed strong collinearity despite chromosomal rearrangements or gene duplications.

### 2.4. Detection of Cis-Acting Elements in the Promoter Regions of SlCPPs

**Figure 4:**
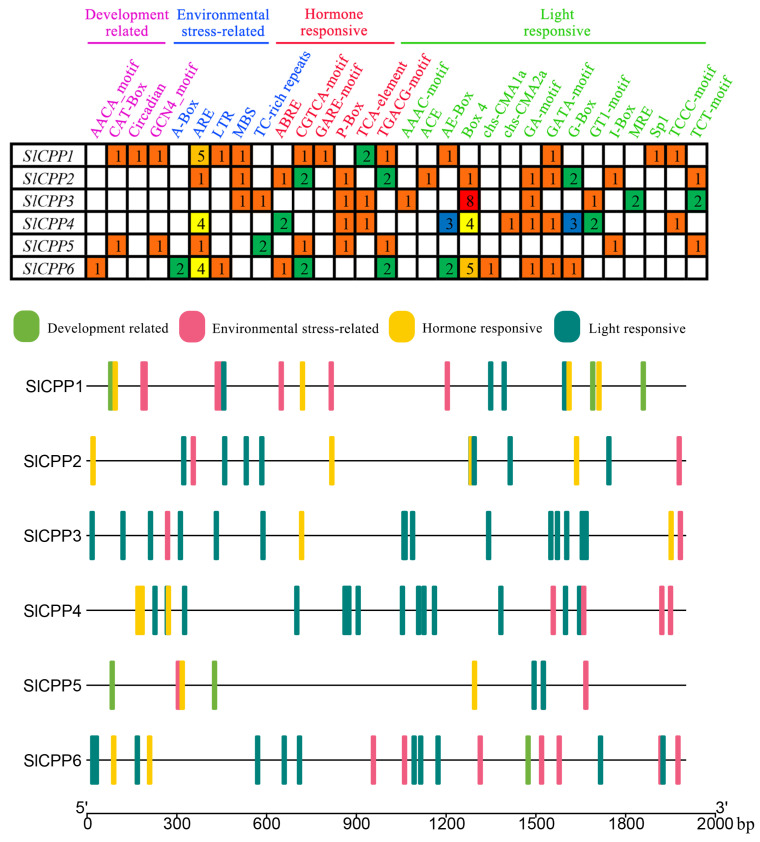
The *cis*-elements in the promoter regions of tomato *SlCPPs*.

Twenty-eight *cis*-acting elements were detected in the promoter regions of the tomato *SlCPPs* (2000 bp upstream of the start codon). These *cis*-acting elements were classified into four categories: development-related, environmental stress-related, hormone-responsive and light-responsive. Details of these *cis*-acting elements are recorded in Figure 4 and Supplementary Table S3. With the exception of *SlCPP3*, all of the *SlCPP* genes contained ARE elements, but *SlCPP3* contained the largest number of Box 4 copies (up to 8). In addition, these *cis*-acting elements included hormone-related elements, jasmonic acid response element, and salicylic acid response element and also cold stress, drought-induced, mechanical injury and anaerobic-induced response elements. These sequence motifs may act as *cis*-elements, putatively participating in hormone-mediated regulation of the promoters.

### 2.5. Prediction of the Tertiary Structure of SlCPP Proteins

**Figure 5:**
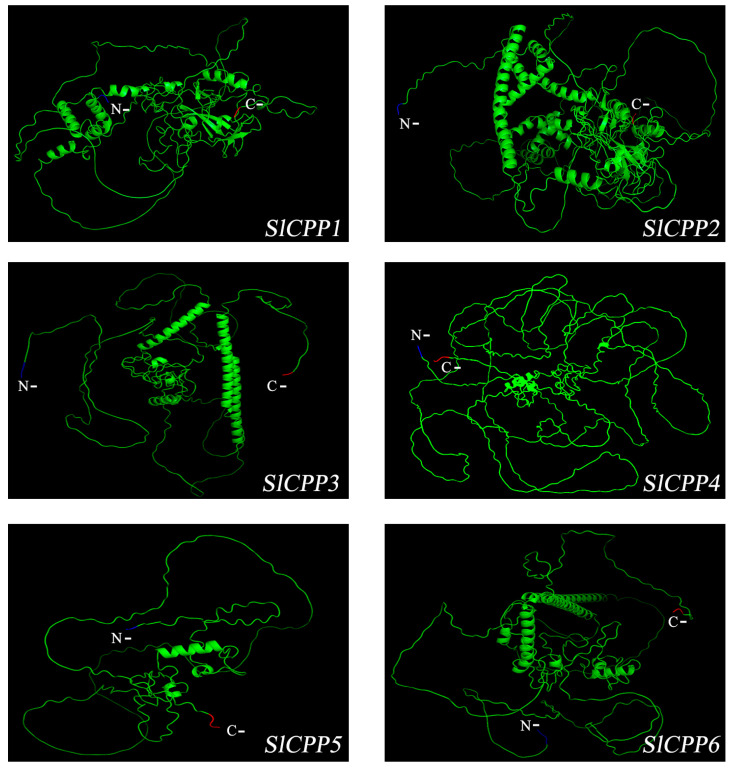
Prediction of the tertiary structure of tomato *SlCPP* proteins. the N- and C- terminal of protein sequence represents the direction of amino acid arrangement during protein biosynthesis.

The tertiary structures of *SlCPP1*-*6* proteins were predicted by AlphaFold2 software based on homology modeling principles. The prediction results showed that the *SlCPP1* and *SlCPP2* proteins have similar structures, with more complex tertiary structure protein structures than the other proteins; the *SlCPP3* and *SlCPP6* proteins have similar structures; and the *SlCPP4* and *SlCPP5* proteins have simpler structures, as detailed in Figure 5. These results provide a good basis for better revealing the functions exercised by CPP proteins in the future.

### 2.6. Expression Patterns of SlCPP Genes Revealed by Transcriptome Analysis

**Figure 6:**
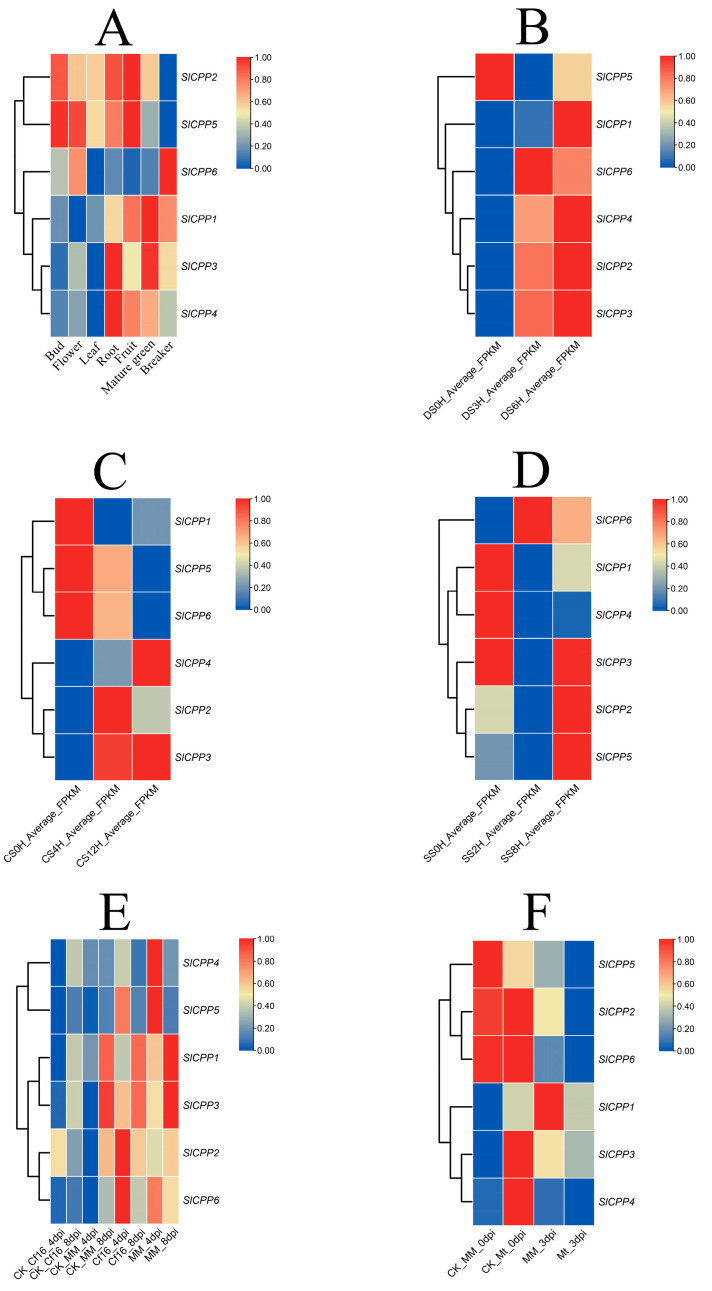
Heatmaps based on RNA-seq data showing the expression profile of the *SlCPPs* in different tomato tissues, and under different abiotic and biotic stress conditions. (**A**) Heatmap of *SlCPPs* expression in tomato plant tissue. (**B**–**D**) Expression patterns of *SlCPPs* in leaves under three abiotic stresses, drought, cold and salt. (**E**,**F**) Expression patterns of *SlCPPs* in leaves inoculated with the pathogenic bacteria *C. fulvum* and *S. lycopersici*. DS: drought stress; CS: cold stress; SS: salt stress; CK: control group.

Analysis of the expression profiles of *SlCPPs* in different tissues of tomato suggested that there were differences in the expression of these genes in different tissues of tomato, but there was no significant specificity, and the expression of *SlCPP1* was higher than other *SlCPP* genes, as shown in Figure 6A and detailed in Supplementary Table S4. These results suggest that the CPP gene family presents a diverse spatiotemporal expression profile in tomato.

In this study, we analyzed the expression patterns of *SlCPP* genes in response to different abiotic and biotic stress treatments, as detailed in Supplementary Table S5. The expression levels in leaves at specific time points under the five tested stresses (drought, cold, salt, inoculation with *C. fulvum* and inoculation with *S. lycopersici*) were compared with the control and finally presented as a heatmap, as shown in Figure 6B–F. After 3 and 6 h of drought treatment, with the exception of *SlCPP5*, the five other *SlCPP* genes were expressed at higher levels than in the control. The expression of *SlCPP5* showed a decrease followed by an increase (Figure 6B). In response to cold stress, there was a decreasing trend in the expression of *SlCPP1*, *5* and *6*, in contrast to an increasing trend in the expression of *SlCPP2*, *3* and *4*, as shown in Figure 6C. After salt treatment, the expression of *SlCPP1* and *4* showed a decreasing trend; the expression of *SlCPP2*, *5* and *6* showed an increasing trend; and the expression of *SlCPP3* showed a decreasing and then increasing trend, as illustrated in Figure 6D. After inoculation with the pathogen *C. fulvum*, there was an overall upward trend in the expression of the *SlCPP* genes (Figure 6E). In response to inoculation with the pathogen *S. lycopersici*, with the exception of *SlCPP1*, the expression of all other *SlCPP* genes showed a decreasing trend, as illustrate in Figure 6F. These results suggest that *SlCPP* genes may play an important role in the response of tomato to abiotic and biotic stresses.

### 2.7. Expression Profiles of SlCPP Genes Analyzed by qRT–PCR

**Figure 7:**
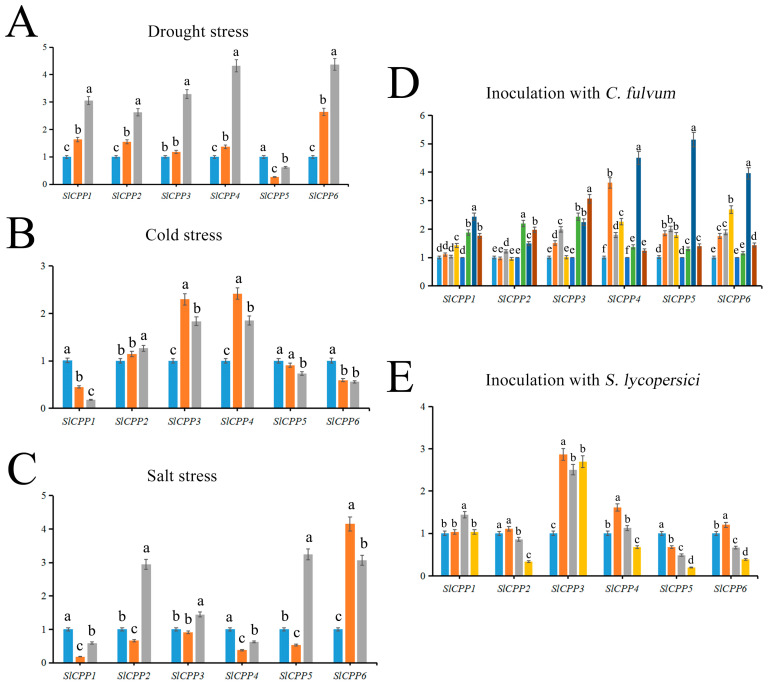
Expression analysis of *SlCPP* genes in tomato under abiotic and biotic stress based on qRT–PCR. Different letters indicate a statistically significant difference (*p* ≤ 0.05), as determined by one-way ANOVA. Error bars indicate ± SD (*n* = 3).

The qRT-PCR results suggested that the transcriptome data were stable and reliable and were also used for the expression pattern exploration of *SlCPPs*, and the detailed data are shown in Supplementary Table S6. None of these genes showed explosive growth term at the time points tested. After drought treatment, with the exception of *SlCPP5*, all five other *SlCPP* genes were differentially upregulated relative to the control (0H), and all peaked at 6 h (Figure 7A). The expression level of *SlCPP6* was the most upregulated, at 4.37 times higher than that of the control. Under cold stress, the expression of *SlCPP1*, *5*, and *6* showed a decreasing trend after treatment, with *SlCPP1* showing the most significant decrease, presenting an expression level 0.18 times that of the control at 12 h after treatment. *SlCPP2*, *3*, and *4* showed upregulated expression levels under cold stress, with *SlCPP4* showing the greatest upregulation, peaking at 2.42 times that of the control at 4 h (Figure 7B). Under salt stress, the expression of most *SlCPP* genes showed a decrease followed by an increase, but the expression of *SlCPP6* peaked at 2 h, at a level 4.15 times higher than that of the control (Figure 7C). Under biotic stress, the expression of *SlCPP* genes showed an elevated trend and was significantly higher than that of the control group after inoculation with the *C. fulvum* pathogen. The expression of *SlCPP5* in Moneymaker plants peaked 4 days after inoculation with the pathogen and was 5.15 times higher than that in the control (Figure 7D). After inoculation with *S. lycopersici*, the expression of *SlCPP1* was slightly upregulated; *SlCPP2*, *4*, *5*, and *6* were downregulated; and *SlCPP3* was significantly upregulated relative to the control group (Figure 7E).

### 2.8. Gene Silencing of SlCPP3 Reduces Drought Resistance in Tomato

**Figure 8:**
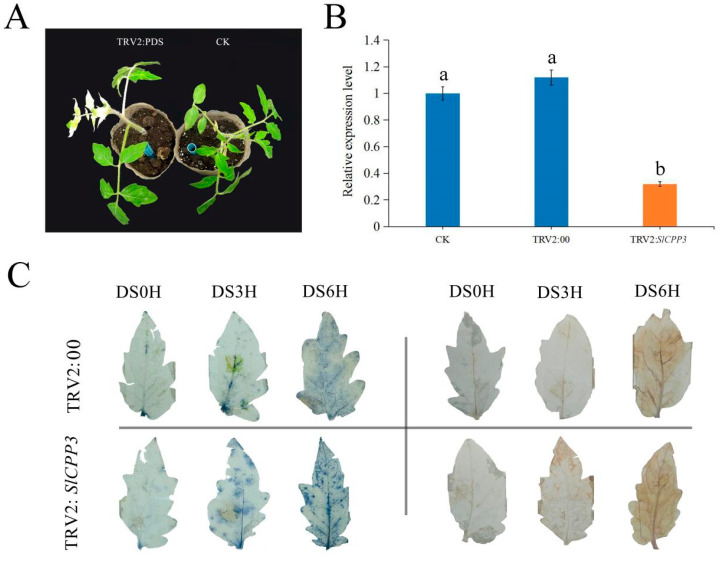
Analysis of *SlCPP3* gene silencing phenotype, silencing efficiency, and detection of reactive oxygen species accumulation after drought treatment. (**A**) Albino plants after gene silencing of *PDS* gene. (**B**) Relative expression of *SlCPP3* gene. Different letters indicate a statistically significant difference (*p* ≤ 0.05), as determined by one-way ANOVA. Error bars indicate ± SD (*n* = 3). (**C**) Accumulation of H_2_O_2_ and O_2_^−^ under drought stress in TRV2:00 and TRV2:*SlCPP3* plants.

After qRT-PCR analysis of gene expression of the *SlCPP* gene family under different treatments, we selected *SlCPP3* as the target gene for gene silencing studies. The optimal region selection for gene silencing sequences is shown in Supplementary Figure S2. After *Agrobacterium*-mediated induction, the tomato plants showed photobleaching on the 15th day of normal incubation (Figure 8A). The gene expression profile of *SlCPP3* showed significantly lower expression in the gene silenced plants than in the control (Figure 8B). The expression of other *SlCPP* genes and predicted genes that may be affected were also examined, and none of them showed significant differences in expression, indicating that *SlCPP3* gene silencing can be used for the next experiments. The results of tomato leaf staining showed that with the prolongation of the simulated drought, the more severe the stress on the plant, the more the accumulation of reactive oxygen species, and the darker the leaves were stained (Figure 8C). Compared with TRV2:00 plants, TRV2:*SlCPP3* plants were more severely stressed, with more reactive oxygen species accumulation and higher staining in the leaves.

### 2.9. Expression of SlCPP3 in the Nucleus

**Figure 9:**
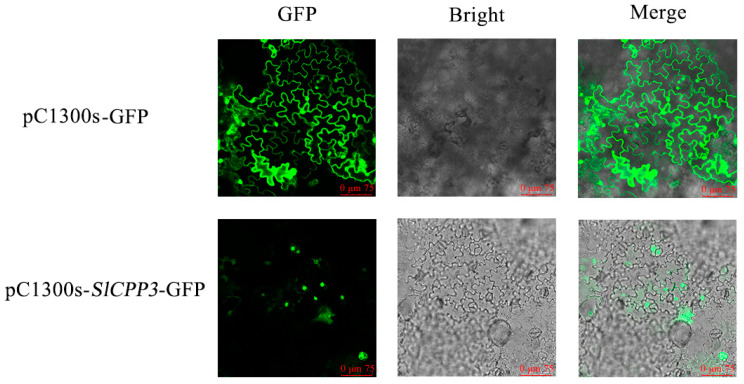
Fluorescence of *SlCPP3* in tobacco leaf cells.

As shown in Figure 9, pC1300s-GFP (empty vector) exhibited a strong green fluorescence effect and the fluorescence of pC1300-*SlCPP3*-GFP appeared only on the nucleus, which confirmed that *SlCPP3* was expressed in the nucleus. This is consistent with the predicted results of subcellular localization.

### 2.10. Analysis of SlCPP3 Gene Expression Network

**Figure 10:**
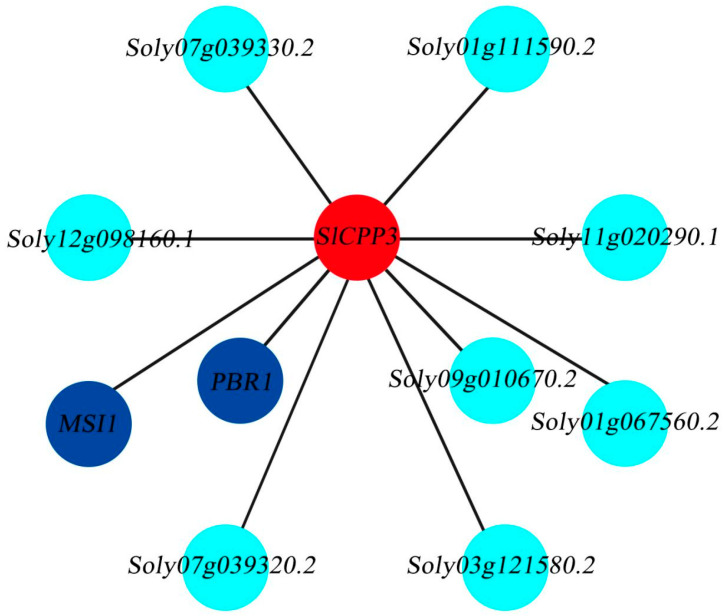
Regulatory network relationships of *SlCPP3* in tomato.

The predicted results of *SlCPP3* interaction network relationship suggested that *SlCPP3* has interaction with many genes, among which there are genes with proven functions (RBR1, MSI), in addition to eight genes with unknown functions (Figure 10). These genes have been proved to have varying degrees of effects on the growth, development and disease resistance of other species. We speculate that *SlCPP3* plays an important role in the regulation of abiotic and biotic stress resistance in tomato.

## 3. Discussion

### 3.1. Identification and Physicochemical Properties of CPP Genes in Tomato

The development and popularity of high-throughput sequencing technology has rapidly advanced the sequencing and assembly of whole plant genomes [28]. In September 2019, a PacBio tomato genome scaffold was de novo assembled with Hi-C technology using Bionano and 10× linked reads for validation, and the tomato genome SL 4.0 and annotated ITAG 4.0 versions were released [29]. The new version has fewer unknown bases and more adequate annotation than the previous version. Based on these data, six *SlCPP* genes were identified in the whole tomato genome in this study by bioinformatics analysis based on the characteristic sequences of the CRC structural domain unique to the CPP transcription factor family. The results of physicochemical property analysis showed that all *SlCPP* proteins were hydrophilic proteins, differing from those in other species, suggesting that not all members of the CPP transcription factor family are hydrophilic or hydrophobic proteins and that they may perform different functions in different species [30].

### 3.2. Distribution of the CPP Gene Family in Plants

CPP proteins are widely present in plants and have been identified in several species [19,20,31]. In the present study, the six identified *SlCPP* genes were all located at the anterior or terminal ends of chromosomes, which is consistent with the distribution of CPP gene family members on chromosomes in other species [20]. The phylogenetic analysis divided the CPP gene family into five branches, which is different from the findings of previous studies. In a study on maize, *ZmCPP* genes were divided into four classes [19]. Interestingly, *ZmCPP* genes were not included in branch III in our classification, suggesting that adding more CPP genes refines the observed phylogenetic relationships. Similar to findings in other species, the *SlCPP* genes were divided into four branches and were found to be more closely evolutionarily related to the genes of potato and pepper, which are also in the Solanaceae family.

### 3.3. Plant Evolutionary Relationships and Genetic Structure

In analyses of plant evolutionary relationships, genes with similar structures and conserved motifs generally have similar functions [32,33]. CPP genes are classified into five categories in *Arabidopsis*, rice, maize, and other crops, and they show similar gene structures and functions. In our study, six *SlCPP* genes were classified into four classes, all of which presented large numbers of introns and exons, and the genes included in the same class presented similar intron–exon arrangements. Recent studies suggest that the deletion and acquisition of introns may be important in facilitating the generation of new genes [34,35]. In the analysis of conserved motifs, five motifs were identified in six *SlCPP* genes, and Motif 1 and Motif 2 were present as typical CPP conserved motifs in all *SlCPP* genes. Within each branch, some motifs are specific, which is the basis for gene family classification and functional differentiation [36,37].

### 3.4. Gene Duplication Events and Synteny Relationships

Gene duplication events are an important mechanism whereby plants evolve, and the membership of their gene families is expanded [38,39]. Previous studies have shown that most plant species have experienced gene duplication or polyploidy events at one time or another [40,41]. No *SlCPP* gene replication events were found in tomato, probably because the CPP gene family has so few members that the probability of a replication event is lower than in other gene families. In studying the phylogenetic relationships of *SlCPP* genes in tomato with those of other plants, we constructed synteny relationships between tomato and *Arabidopsis* and potato. Finally, four pairs of syntenic CPP genes were identified between tomato and *Arabidopsis*, and six pairs were identified between tomato and potato. These results indicate that the CPP genes of different species are linked, and that the homology between tomato and potato is greater than that between tomato and *Arabidopsis,* which further indicates that the evolutionary distance between tomato and potato is shorter [42].

### 3.5. The Promoters of SlCPP Genes Contain Many Cis-Acting Elements

*Cis*-acting elements in promoter regions can specifically bind to transcription factors to initiate the expression of downstream genes [43]. As a result of the analysis of *cis*-acting elements in the promoter regions of *SlCPP* genes, we identified a large number of *cis*-acting elements in the promoter regions of these *SlCPP* genes that are related to plant growth and development and resistance to the adverse external environment. Hormone-related *cis*-acting elements (CGTCA motif, ABRE, P-box, TCA element and TGA element) are also abundantly present [44,45,46]. The presence of these various *cis*-acting elements in their promoter regions allows the *SlCPP* genes to play extraordinary roles in the regulation of normal plant growth.

### 3.6. Tertiary Structure of SlCPP Proteins

In the most recent generation of algorithms of the DeepMind team, AlphaFold 2, has emerged [47,48]. The algorithm is able to accurately predict the tertiary structure of proteins based on amino acid sequences with an accuracy comparable to that of the tertiary structures resolved using experimental techniques such as cryoEM, NMR or X-ray crystallography [49]. We applied this technique to the structure prediction of *SlCPP* proteins and achieved satisfactory results. From these tertiary structure diagrams, we can conclude that the treater the number of motifs present in a protein, the more complex its structure will be, and that the coiling and folding of these proteins are closely related to the gene structure. *SlCPP1* and *SlCPP2* belong to the same branch, and their protein tertiary structure are similar and more complex than those of other *SlCPP* proteins. We speculate that the structural differences in these proteins lead to functional differences between them.

### 3.7. Transcriptomics Combined with qRT–PCR Reveals the Expression Profile of SlCPP Genes in Tomato

Many studies have shown that CPP transcription factors are both associated with plant growth and development and able to respond to hormone induction and abiotic and biotic stresses [19,24]. For example, evidence from a *TSO1* mutant demonstrates that this gene regulates shoot and root differentiation and inflorescence development in *Arabidopsis* [23]. The resolution of tomato *SlCPP* gene transcriptome data from different tissues of tomato revealed that *SlCPP* transcription factors were generally highly expressed in roots.

Drought stress induces decreases in leaf stomatal conductance and water loss in plants, which helps to maintain the intracellular water status of plants under water deficit conditions [50,51]. In this study, a large number of drought-responsive *cis*-elements were identified in the promoter regions of *SlCPP* family members, implying that this family may play a role in drought stress. The qRT–PCR analysis revealed that the expression levels of all six *SlCPP* members were differentially upregulated under drought treatment, which further supported the results of the promoter analysis. A previous study showed that the expression of four members of the maize *ZmCPP* family was significantly upregulated under drought stress induction [19]. In conclusion, these results suggest that *SlCPP* genes play a positive regulatory role in the drought stress response.

In addition, under cold and salt-induced conditions, the expression pattern of *SlCPPs* was similar to that under drought stress, but not all *SlCPP* genes were upregulated. We speculate that these genes may play other important roles in the response to environmental stress. Studies addressing this aspect are still lacking at present, and the specific response mechanisms involved need to be further investigated.

Regarding biotic stresses, we found that the expression of *SlCPP* genes showed an increasing trend under tomato leaf mold pathogen infestation, whereas under gray leaf spot pathogen infestation, *SlCPP* gene expression varied. This suggests that the response patterns and rates of *SlCPP* genes may not be the same under these two different types of pathogen infestation. Under in vivo infestation with the necrotroph *C. fulvum*, *SlCPP* genes play a positive regulatory role. In contrast, the role of *SlCPP* genes related to the biotrophic *S. lycopersici* pathogen is unclear.

### 3.8. The Role of SlCPP3 Gene in Abiotic and Biotic Stress

Virus-induced gene silencing experiments demonstrated that *SlCPP3* was resistant to drought stress, but the effect was not particularly pronounced. Based on the STRING database, we predicted the interaction between *SlCPP3* gene and other genes. *SlCPP3* gene interacts with *RBR1* and *MSI1* genes. *RBR1* gene is involved in plant growth and development and biological stress, and *MSI1* gene is related to plant drought resistance [52,53,54]. We speculate that *SlCPP3* gene plays a role in abiotic and biotic stress in tomato.

## 4. Materials and Methods

### 4.1. Plant Growth and Treatments

The tomato variety Ontario 7816 (resistant to leaf mold, containing the *Cf*-*16* gene), the tomato variety Motelle (resistant to gray leaf spot, containing the *Sm* gene), the tomato varieties ‘Moneymaker’ (susceptible variety) and ‘Micro-Tom’ (common cultivated variety) were conserved in our laboratory. These plants were grown in sterile nutrient soil in an artificial climate chamber (Xuelai Biotechnology, Nanjing, China). The environmental program was set to 16 h of light at 40,000 lx, at 24 °C with 60% relative humidity, and 8 h of darkness at 18 °C with 50% relative humidity.

For the abiotic stress treatments, 4-week-old Micro-Tom seedlings showing uniform growth and health characteristics were selected and transferred to hydroponic conditions for 48 h. Drought stress: drought stress was simulated with a 15% PEG6000 (Coolaber Biotechnology, Beijing, China) solution, and plant leaves were collected at specific time points (0, 3 and 6 h); Cold stress: the experimental plants were transferred to a constant temperature growth chamber maintained at 5 °C, and their leaves were collected at specific time points (0, 4 and 12 h); Salt stress: seedlings were transferred to a 200 mM sodium chloride (NaCl) solution (Coolaber Biotechnology, Beijing, China), and the leaves were collected at specific time points (0, 2 and 8 h) [55]. Each treatment group included 30 tomato seedlings, and the whole experiment was repeated three times [55,56]. For the biotic stress treatments, 30 tomato seedlings of varieties with resistance or susceptibility to the selected pathogens showing uniform growth were selected for each group at 4 weeks of age [57]. The whole plants were sprayed with 50 mL of a suspension of pathogenic spores containing either 1 × 10^7^ spores/mL of *C. fulvum* or 1 × 10^4^ spores/mL of *S. lycopersici* [57,58]. Leaves of these plants were collected at specific time points (gray mold: 4 and 8 days, gray leaf spot: 0 and 3 days). The collected leaves were rapidly frozen in liquid nitrogen. The whole experiment was repeated three times.

### 4.2. Identification of CPP Gene Family Members in Tomato

The Hidden Markov model (HMM) profile data of CXC (PF03638) domains (http://pfam.xfam.org/, accessed 15 February 2022) were used for CPP gene family member identification with reference to the method of Sun et al. [59].

### 4.3. Bioinformatics Analysis of CPP Gene Family in Tomato

The evolutionary relationships of CPP gene families in tomato and other species (*A. thaliana*, rice (*Oryza sativa*), pepper (*Capsicum annuum*), etc.) were analyzed with reference to the method of Yang et al. [60]. *SlCPPs* gene sequences, homology analysis, and *cis*-acting element analysis were referenced to the methods of Sun et al. [61].

### 4.4. Tertiary Structure Prediction of SlCPP Proteins

The tertiary structure prediction of *SlCPPs* was performed using AlphaFold2 software (module version) built on the π 2.0 supercomputing platform of Shanghai Jiao Tong University [62]. The highest pLDDT values among the five obtained models were selected as the final result. The tertiary structure visualization of *SlCPP* proteins was performed with PyMOL 2.5 software (https://pymol.org/2/, accessed on 4 March 2022).

### 4.5. Analysis of SlCPP Expression Patterns Based on Transcriptomic Data

The transcriptomic data of different tissues of the tomato variety Heinz 1706 were downloaded from the Tomato Functional Genomic Database (http://ted.bti.cornell.edu/cgi-bin/TFGD/; NCBI accession number SRA049915, accessed on 16 February 2022) [59]. Abiotic stresses: the raw transcriptome data of the treated Micro-Tom tomatoes have been uploaded to NCBI. The corresponding accession numbers for the drought, cold and salt treatments are PRJNA624892, PRJNA626343 and PRJNA624032, respectively. Biological stress: the raw transcriptome data of tomato plants treated with sprayed pathogens are stored in the NCBI database. The registration numbers of the tomato gray mold- and gray leaf spot-related transcriptome data are PRJNA552220 and SRP097450, respectively.

### 4.6. qRT–PCR Analysis of SlCPPs Expression

Total RNA was extracted using the Total Plant RNA Extraction Kit (ProMag, Beijing, China, code no. LS1040). The integrity of the total RNA was examined by 1.00% agarose gel electrophoresis. The purity and concentration of total RNA were determined by measuring OD260/280 values with an Eppendorf BioSpectrometer UV/Vis spectrophotometer (Eppendorf, Hamburg, Germany). After the RNA concentration was adjusted, the reverse transcription reaction was performed according to the instructions of the PrimeScript^TM^ 1st Strand cDNA Synthesis Kit (TaKaRa, Shiga, Japan). Specific primers for *SlCPPs* were designed with Primer Premier 5.0 software, as described in Supplementary Table S7. After the comparative analysis of the stability of several housekeeping genes under different treatment conditions, *β-Actin* was used as an internal control [63]. qRT-PCR was performed with three independent biological replicates using AceQ^®^ qPCR SYBR^®^ Green Master Mix (Vazyme, Nanjing, China) in 20 microliter volume on a qTOWER3G Real-time System (Analytik Jena AG, Jena, Germany). The relative expression levels of *SlCPP* genes were calculated using the 2^−ΔΔCT^ algorithm [64].

### 4.7. VIGS Vector Construction and Agroinfiltration

The first step was the selection of the target gene sequence fragment, and we used the SGN VIGS Tool to intelligently select the best silencing fragment (https://vigs.solgenomics.net/, accessed 7 May 2021). Next, the target fragment was ligated to the qTRV2 vector. Subsequently, the recombinant vector was introduced into tomato seedlings by *Agrobacterium*-mediated method [65]. Finally, the gene silencing effect was quantified by gene expression.

### 4.8. Observation of Stained Tissue

The accumulation of H_2_O_2_ and O^2−^ in TRV2: *SlCPP3* and TRV2: 00 plant leaves was detected by 3,3′-diaminobenzidine (DAB) and nitrotetrazolium blue chloride (NBT) (Coolaber Biotechnology, Beijing, China) staining under drought stress treatment, respectively [66].

### 4.9. Subcellular Localization of SlCPP3

Firstly, the full-length CDS of *SlCPP3* gene was ligated with pCAMBIA1300s-*GFP* vector. Subsequently, the recombinant vector was transformed into tobacco seedlings by *Agrobacterium*-mediated method [67]. Finally, the leaves of tobacco seedlings cultured under low light conditions for 2 days were placed under a laser confocal microscope to observe the fluorescence phenomenon.

### 4.10. Analysis of the Expression Network of SlCPP3 in Tomato

The key genes related to abiotic stress and biotic stress in tomato were identified by selecting *SlCPP3* through database resources. The *SlCPP3* expression network was constructed by referring to the method of Sun et al. [61].

## 5. Conclusions

The sequencing and assembly of the most recent tomato whole genome, which has already been deeply annotated, have made it possible to study the functional characteristics of the CPP gene family at the genomic level. In this study, six *SlCPP* genes were identified for the first time using the most recent tomato whole genome, and the physicochemical properties, phylogenetic relationships, gene structural features, synteny relationships and *cis*-acting elements of the *SlCPP* genes were systematically and comprehensively analyzed. We used the most recent protein tertiary structure prediction system, AlphaFold2, to predict the tertiary structures of these genes. Transcriptomics combined with qRT–PCR was used to analyze the expression patterns of *SlCPP* genes under abiotic and biotic stresses, and the results showed that *SlCPP* genes play roles in the responses to three abiotic stresses, drought, cold and salt, and positively regulate the infestation of necrotrophic *C. fulvum* pathogens. Virus-induced gene silencing demonstrates that *SlCPP3* is resistant to drought stress. Finally, *SlCPP3* was predicted to interact with 10 genes, including *RBR1* and *MSI1* using the STRING database. The specific functions of CPP gene family members in tomato remain to be investigated, and the current results provide a theoretical basis for further studies on the functions of *SlCPP* genes in tomato.

## Data Availability

The raw sequencing data of this article are stored in the NCBI Sequence Read Archive under accession number SRA049915, PRJNA624892, PRJNA626343, PRJNA624032, PRJNA552220 and SRP097450.

## Supplementary Materials

The following supporting information can be downloaded at: https://www.mdpi.com/article/10.3390/ijms24065762/s1.
