# Supplementary material for: Genome-Wide Identification and Expression Analysis of Cysteine-Rich Polycomb-like Protein (CPP) Gene Family in Tomato
Source: Int J Mol Sci. 2023 Mar 17;24(6):5762. doi: 10.3390/ijms24065762 (PMC10058331; doi:10.3390/ijms24065762)
Supplement: Supplementary file 1 [file ijms-24-05762-s001.zip › ijms-2235528-supplementary/All Supplementary Materials/Table S1.pdf]

Supplementary Table S1: Gene descriptions and protein characteristics of 6 members of CPP gene family in tomato.

Gene name	Gene ID	CDS length (bp)	Chr	Position（5'-3'）	Protein length (aa)	Protein characteristics					Subcellular location
						MW (kDa)	Theoretical pI	Instability Index	Aliphatic Index	GRAVY	
SlCPP1	*Solyc01g079390.3*	1734	1	78333325-78341795	577	64.60	8.43	42.54	59.36	-0.793	Nucleus
SlCPP2	*Solyc03g044380.3*	2766	3	8971764-8981370	921	102.90	8.42	52.74	64.60	-0.767	Nucleus
SlCPP3	*Solyc07g020710.3*	1713	7	13274624-13291578	570	61.99	7.48	48.30	70.54	-0.609	Nucleus
SlCPP4	*Solyc08g067150.3*	2922	8	56136445-56144614	973	104.73	6.14	57.47	69.11	-0.591	Nucleus
SlCPP5	*Solyc09g082505.1*	915	9	68646637-68648720	304	33.02	8.53	66.02	59.08	-0.553	Nucleus
SlCPP6	*Solyc12g007180.2*	1857	12	1616338-1626340	618	67.06	8.19	52.32	62.36	-0.618	Nucleus

Abbreviations: MW, Molecular weight; GRAVY, Grand average of hydropathicity.
