# Supplementary figures and images for: Genome-Wide Identification and Expression Analysis of Cysteine-Rich Polycomb-like Protein (CPP) Gene Family in Tomato
Source: Int J Mol Sci. 2023 Mar 17;24(6):5762. doi: 10.3390/ijms24065762 (PMC10058331; doi:10.3390/ijms24065762)

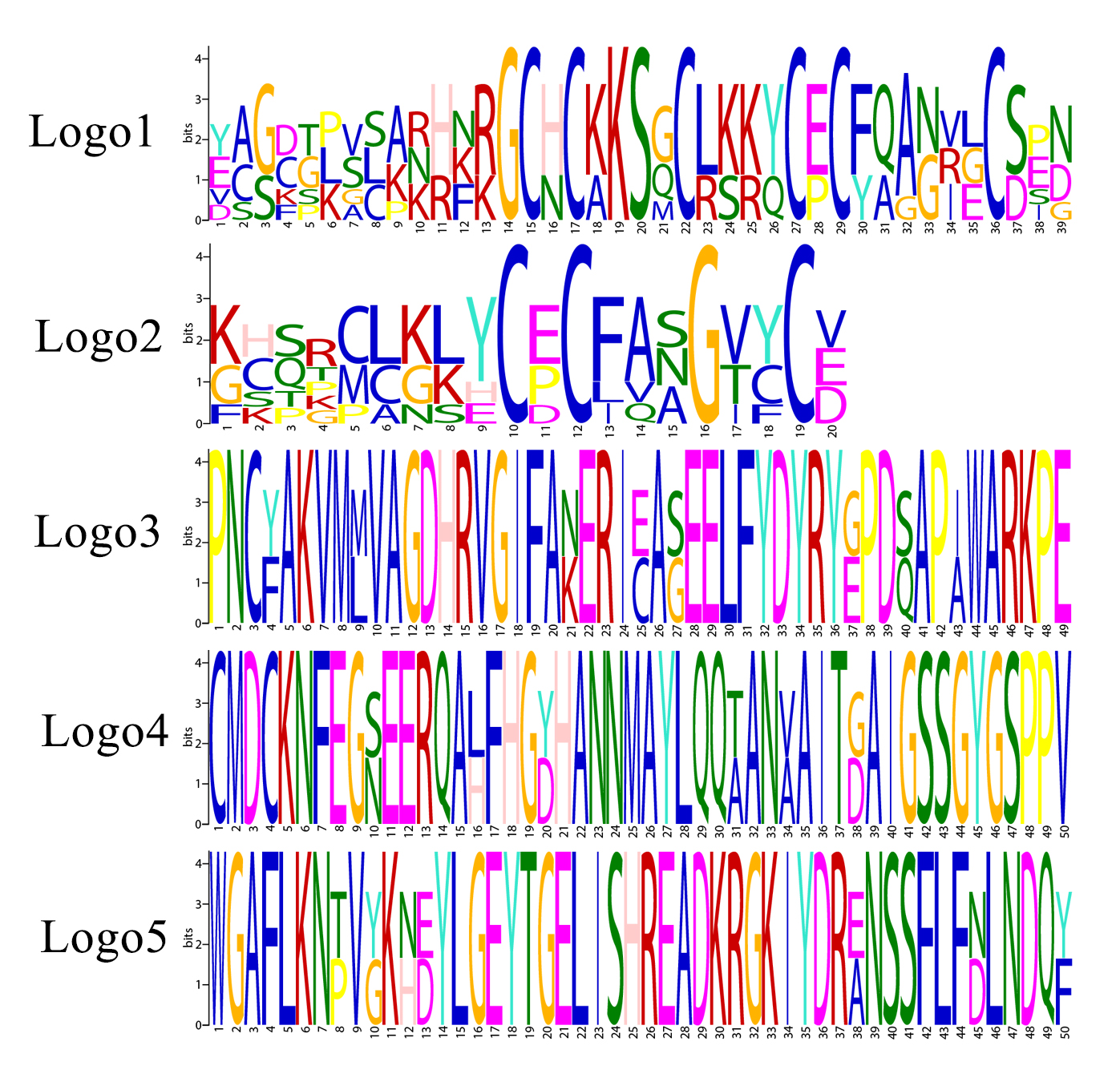

Supplement: Supplementary file 1 [file ijms-24-05762-s001.zip › ijms-2235528-supplementary/All Supplementary Materials/Figures S1 Logo details for all conservative motifs in SlCPPs.jpg]

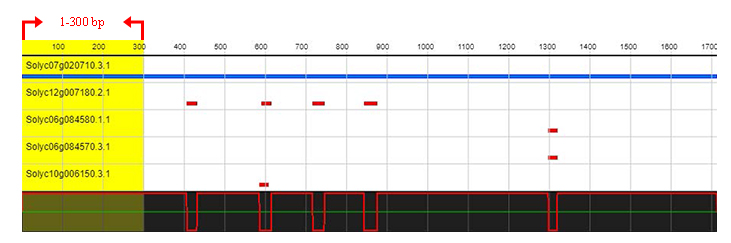

Supplement: Supplementary file 1 [file ijms-24-05762-s001.zip › ijms-2235528-supplementary/All Supplementary Materials/Figures S2 Selection of optimal gene silencing sequence regions for SlCPP3.jpg]
